# Quorum Sensing and Quorum Quenching with a Focus on Cariogenic and Periodontopathic Oral Biofilms

**DOI:** 10.3390/microorganisms10091783

**Published:** 2022-09-03

**Authors:** Patricia P. Wright, Srinivas Sulugodu Ramachandra

**Affiliations:** 1 School of Dentistry, The University of Queensland, 288 Herston Road, Herston, QLD 4006, Australia; 2 Dental Health Clinic, BC Road, Dakshina Kannada, Bantval City 574219, India

**Keywords:** autoinducers, cariogenic biofilms, *Fusobacterium nucleatum*, periodontopathic biofilms, quorum sensing, quorum quenching, red complex pathogens, *Streptococcus mutans*

## Abstract

Numerous *in vitro* studies highlight the role of quorum sensing in the pathogenicity and virulence of biofilms. This narrative review discusses general principles in quorum sensing, including Gram-positive and Gram-negative models and the influence of flow, before focusing on quorum sensing and quorum quenching in cariogenic and periodontopathic biofilms. In cariology, quorum sensing centres on the role of *Streptococcus mutans*, and to a lesser extent *Candida albicans*, while *Fusobacterium nucleatum* and the red complex pathogens form the basis of the majority of the quorum sensing research on periodontopathic biofilms. Recent research highlights developments in quorum quenching, also known as quorum sensing inhibition, as a potential antimicrobial tool to attenuate the pathogenicity of oral biofilms by the inhibition of bacterial signalling networks. Quorum quenchers may be synthetic or derived from plant or bacterial products, or human saliva. Furthermore, biofilm inhibition by coating quorum sensing inhibitors on dental implant surfaces provides another potential application of quorum quenching technologies in dentistry. While the body of predominantly *in vitro* research presented here is steadily growing, the clinical value of quorum sensing inhibitors against *in vivo* oral polymicrobial biofilms needs to be ascertained.

## 1. Introduction

Quorum sensing allows bacteria in biofilms to chemically communicate in order to respond to environmental changes by coordinating their activity as if they were multicellular organisms [1]. Bacteria synthesize and export signalling molecules called autoinducers (AIs) [2,3]. At a particular threshold or “quorum” concentration of extracellular AI molecules, biofilm bacteria perceive the existence of these signalling compounds, resulting in changes in gene expression, and causing behavioural changes in biofilms. A cellular cascade is activated simultaneously in many bacteria which benefits the bacterial population as a whole by permitting the en masse expression of various virulence factors [4]. The property of behavioural change in biofilms due to altered gene expression because of the accumulation of AIs is known as quorum sensing (QS).

The phenomenon of QS was discovered over 50 years ago with the observation that bioluminescence in the marine bacteria *Photobacterium fischeri* occurred only at an increased cell density [5]. Since then, QS has been shown to be involved in the regulation of several biofilm processes including, bioluminescence, virulence, competence, conjugation, motility, and biofilm formation [6]. Various stages of biofilm development, including microcolony formation, during which extracellular polymeric substance (EPS) is secreted [7], and biofilm maturation and dispersal, are regulated by QS [8,9]. The initiation of QS can also result in the elimination of non-competent bacteria by competent bacteria, resulting in a highly virulent biofilm community [10].

Biofilm bacteria are enmeshed in a self-produced matrix of EPS, causing a many-fold increase in the resistance to the action of antimicrobials and to host immune defences compared to planktonic bacteria [11]. This feature of biofilms is becoming increasingly problematic because many chronic infections in humans are biofilm-related and are treated with antimicrobials [12]. Of particular concern is the indiscriminate overuse of antimicrobials which may result in the development of antimicrobial resistance (AMR) [13]. The rise of AMR is recognized as a major challenge currently faced by health care [13,14]. The problem is compounded by several factors including over-prescription, self-prescription and in certain countries, the unregulated prescription of antibiotics. Furthermore, the guidelines for antimicrobial stewardship are either non-existent or inconsistent [15], and pharmaceutical companies are reluctant to invest in novel antimicrobial research [16]. Additionally, a huge percentage of antimicrobials are prescribed for livestock growth promotion and in aquaculture [17,18]. Thus, researchers and clinicians have been forced to explore new ways to treat biofilm-mediated diseases. One such approach is quorum quenching (QQ) [19].

Oral diseases, including caries and periodontitis, are due to microbial dysbiosis [20]. Establishing health in such instances depends on rebiosis or establishing eubiosis [21]. However, a dysbiotic microbial flora is extremely resistant to change as it exists as a climax community. Efforts to establish eubiosis by mechanical debridement may result in short-term gains with a high chance of disease recurrence as microbial dysbiosis re-establishes [22]. To avoid disease recurrence, and to enhance the longevity of gains obtained by mechanical therapy, in some instances mechanical therapy is combined with systemic antimicrobials [22]. However, with the ever-increasing risk of the development of antimicrobial resistance, alternative therapeutic approaches need to be sought [16]. One such approach is to employ a variety of natural products such as phytochemicals [23]. The mechanism of action of specific natural products may not always be clear, but in some instances, they are known to be effective due to QS inhibition [24,25,26]. Indeed, further developing a knowledge of QS inhibition or QQ provides the opportunity to offer treatment modalities from a variety of sources including plants, bacteria, and synthetic products, free from issues of antimicrobial resistance.

QQ, which aims to interrupt bacterial communication networks responsible for virulence, has several possible applications in dentistry. Untreated dental caries in the permanent dentition accounts for 17% of global production loss [27] and represents the most prevalent condition identified in an analysis of the worldwide burden of disease [28]. Similarly, the global burden of periodontitis is enormous, and the disease has been associated with several medical conditions [29,30]. It is of little surprise then, that in recent years, research has sought alternative antimicrobial approaches [31,32], and in particular QQ has featured highly as a possible novel treatment for dental disease [26,33,34]. This narrative review aims to explain basic concepts in QS, including Gram-negative and Gram-positive intraspecies bacterial models, interspecies QS, QS in the fungus *Candida albicans*, and the influence of flow on QS. A further aim is to summarize the current literature regarding QS and QQ in cariogenic and periodontopathic biofilms, and to explore specific applications of QQ in implant dentistry.

## 2. Bacterial QS Model Systems

There are three basic systems of QS in bacteria. At an intraspecies level, Gram-negative bacteria communicate via AIs called acyl-homoserine lactones (AHLs), while in Gram-positive organisms AIs in the form of oligopeptides mediate cell-to-cell signalling. The third system for interspecies signalling, common to both Gram-positive and Gram-negative organisms, is based on a derivative of 4,5-dihydroxy-2,3-pentanedione (DHPD) called Autoinducer-2 (AI-2) [35].

### 2.1. Intraspecies QS in Gram-Negative Bacteria

**Figure 1:**
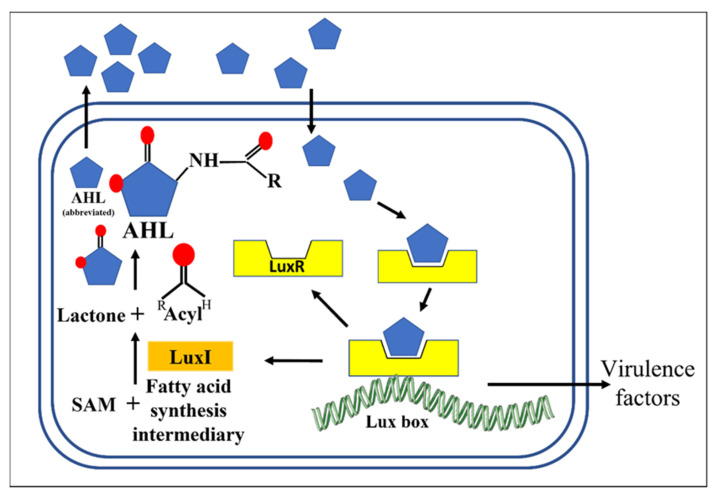
QS model in Gram-negative bacteria. Feedback loop involving the catalysis of acyl-homoserine lactone (AHL) by LuxI from S-adenosylmethionine (SAM) and fatty acid synthesis intermediaries. Upon re-entry into the cell, AHL combines with LuxR to initiate the expression of virulence factors, LuxI and LuxR.

The Gram-negative model is based on the lux regulon. The word “lux” indicates the luminescence gene [36]. In 1981, an AHL was identified as the AI for *Photobacterium fischeri* [37], followed by the identification in another marine species, *Vibrio fischeri*, of LuxR and LuxI as QS mediators. LuxR is a protein regulator and LuxI is an enzyme essential to the biosynthesis of AHL [38,39]. AHL, when in a complex with LuxR, is a transcription factor which binds to the lux regulon at a series of nucleotides known as the lux box. Binding initiates the expression of LuxI, LuxR, and proteins involved not only in bioluminescence in certain bacteria but also in bacterial virulence in particular pathogens [40]. Many Gram-negative bacteria from the phylum proteobacteria, including the well-studied pathogen *Pseudomonas aeruginosa*, conform to this model, each employing LuxI/LuxR homologues and their own specific AHL for QS [41]. The model is described in Figure 1.

### 2.2. Intraspecies QS in Gram-Positive Bacteria

**Figure 2:**
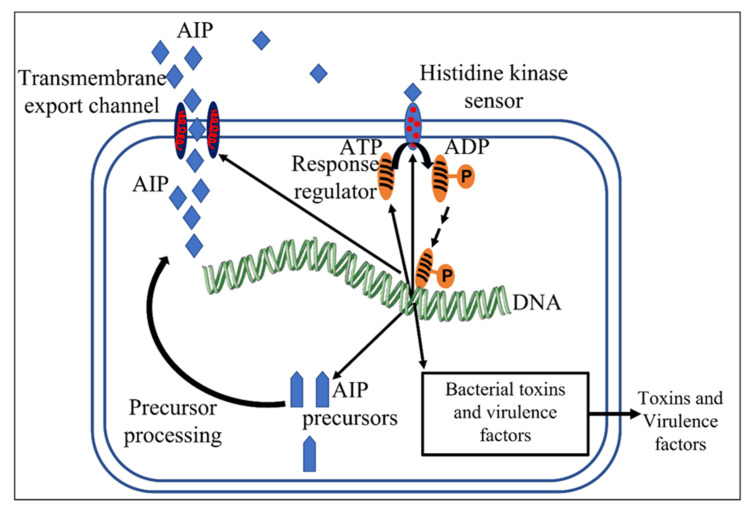
QS in Gram-positive bacteria. AIP (autoinducing peptide) is exported extracellularly, where at increased cell density, enough accumulates to be sensed by the histidine kinase receptor to initiate the transcription of AIP precursors, virulence factors, an export channel, and the regulator and receptor proteins.

The Gram-positive model resembles the Gram-negative model to some degree, in that in pathogens, the products of gene transcription result in increased bacterial virulence and produce the components needed to maintain a positive feedback loop. However, the components are specific to Gram-positive bacteria [3]. They include an autoinducing peptide (AIP) precursor, a transmembrane transport protein channel to export AIP into the extracellular environment, and a sensor histidine kinase that initiates signal transduction by causing the ATP mediated phosphorylation of a response regulator protein which then acts as a transcription factor [3]. The model is described in Figure 2.

### 2.3. Interspecies AI-2 Signalling Model

AI-2 is widely used by Gram-positive and -negative bacteria for interspecies communication [42]. AI-2 synthesis depends on an enzyme called LuxS which catalyses the conversion of S-ribosylhomocysteine to DHPD and homocysteine in the final step of an important bacterial pathway responsible for the methylation of proteins and nucleic acids [43]. AI-2 is a molecule that binds ribose-like structures [40]. This point is of particular relevance, as seen in Section 5.2, where sugars including D-ribose act as QS inhibitors [44]. A structural study revealed that the AI-2 in *Vibrio. harveyi* is a bicyclic borate derivative of DHPD, a molecule capable of adopting different cyclic configurations, depending on its environment [45]. In other bacteria, for example in the species *Salmonella typhimurium*, AI-2 is a monocyclic form of DHPD which does not contain boron [46].

As AI-2 accumulates extracellularly, a periplasmic receptor binds to AI-2 to initiate signal transduction that in turn enables gene transcription and virulence. In *V. harveyi*, this receptor protein is LuxP [45]. In several proteobacteria, the receptor protein is LsrB, which is part of an ABC transporter designed to enable AI-2 entry into the cell [40]. LsrB is transcribed from the lsr operon in response to AI-2 [40]. Recently, a third receptor was identified in *Pseudomonas aeruginosa* where the receptor is comprised of chemotactic and calcium channel components [47].

## 3. The Effect of Fluid Flow in QS

Because the process of QS occurs through AI signalling molecules which diffuse into the surrounding environment, the concentration of these signalling molecules is dependent on fluid flow [48]. Static biofilm models are still commonly encountered in *in vitro* research [49], although, in nature, most biofilms exist with fluid flowing in their surroundings. Thus, one of the focuses of QS research in biofilms is fluid flow. Experimental and modelling studies have demonstrated that, in a *Pseudomonas aeruginosa* biofilm, as the flow rate increased, higher biofilm biomass was needed to induce QS [50]. Interestingly, in some instances, an increased flow rate improves QS, whereas in other circumstances QS decreases [50]. The relationship of the flowing liquid to the biofilm is important, with QS repression in the bacteria at the biofilm–fluid interface, and QS activation in the deep biofilm layers [51]. As a similar concept, it has been demonstrated that, by a positive feedback mechanism in QS networks, bacterial biomass overcomes the gradients created by fluid flow [48]. This allows biofilms to exhibit QS-like behaviour despite fluid flow [48].

## 4. QS in Cariology

Dental carious lesions are associated with salivary factors such as lowered pH, causing tooth demineralization, and decreased salivary calcium and phosphate ion concentrations which are insufficient to remineralize the tooth [52]. Acidic saliva is largely linked to the presence of the acidogenic bacterial species *Streptococcus mutans* [53]. However, other oral pathogens including *Streptococcus sobrinus* and *Streptococcus parasanguinis*, bacteria of the genera *Veillonella* [53], *Actinomyces* and *Prevotella* [54], and the fungus *Candida albicans*, play contributory roles in carious biofilm formation [55]. In part, the dominance of *S. mutans* in the aetiology of dental caries helps explain the extensive QS research that exists for this species. The goal of much of this work is to identify QS inhibitors capable of attenuating dental caries [26,56,57]. From another perspective, interest is centred on *S. mutans* because of its role as a model organism for studying crosstalk between QS networks [58].

**Figure 3:**
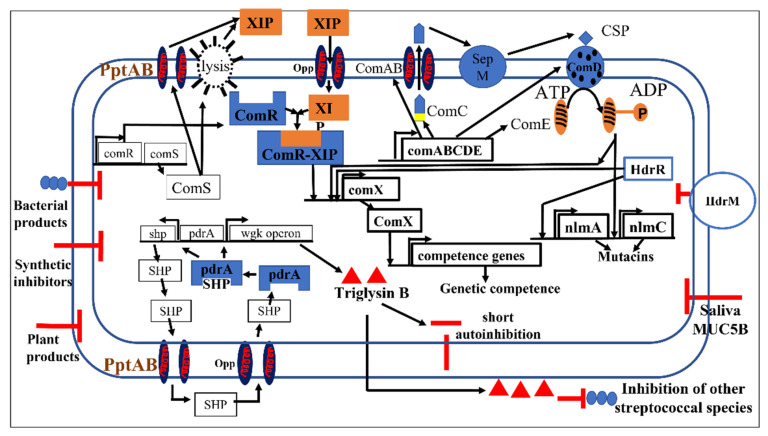
The CSP/ComABCDE, XIP-ComRS and PdrA-WGK pathways and their interaction, inhibition, and activation in *S. mutans*.

QS in *S. mutans* controls biofilm formation [34,59], genetic competence and the production of bacteriocins called mutacins [60,61,62]. Genetic competence permits *S. mutans* to transform its genome by the incorporation of other microorganisms’ DNA, while bacteriocins target competing bacteria to kill or inhibit them [63]. The two well-studied and interconnected QS intraspecies pathways for communication between *S. mutans* cells are the competence stimulating peptide (CSP) system, also known as ComABCDE, and the XIP-ComRS signalling system [10,58,63]. Recently, a further QS pathway, the PdrA/WGK system, was identified in *S. mutans* [64]. These three signalling systems, described in greater detail in Section 4.1.1, Section 4.1.2 and Section 4.1.3 are summarized in Figure 3.

Interspecies communication via the AI-2-LuxS system is also well documented for *S. mutans* biofilms [26,65,66]. Additionally, *S. mutans* biofilms can be attenuated by QS involving several species of commensal streptococci [67]. While the last decade has seen considerable advances in the knowledge of QS in *S. mutans*, it is thought that other signalling peptide systems are yet to be discovered [58].

The yeast *C. albicans* also plays a contributory role in dental caries. While this eukaryote is well-known for its role in the mucosal lesions of the oral candidiasis [68], its presence is positively associated with early childhood caries (EEC) [55]. *Candida* species are considered to be important in the maintenance of dentinal carious lesions [69], and are twice as likely to be found in the saliva of children with severe ECC compared to caries-free children [70]. In 2001, *C. albicans* was found to produce a QS molecule called farnesol that prevents the yeast to hyphae transition [71]. Since then, QS and QQ mechanisms have been explored in *C. albicans* centred on farnesol, amongst others. These are discussed in detail in Section 4.2.

### 4.1. QS and QQ in S. mutans

Understanding the QS networks in *S. mutans* provides the opportunity to utilize QQ as an alternate approach to the prevention of dental caries, by targeting signalling networks and the pathogenicity of *S. mutans*. Typically, experimental studies are performed *in vitro* and employ reverse transcription and polymerase chain reaction assays to demonstrate the downregulation of genes involved in QS. This is accompanied by phenotypic change such as a decrease in biofilm volume, hydrophobicity or EPS [26,34,59]. Some studies provide further evidence of QQ by the inclusion of assays demonstrating reductions in genetic transformation and bacteriocin production [56,59].

The CSP-ComABCDE, XIP-ComRS and the interspecies AI-2/LuxS pathways are all targets for QQ. Interference with the AI-2/LuxS pathway is usually studied using mutant strains deficient in luxS [65,66]. In the case of biofilms of ΔluxS *S. gordonii* coaggregates with *S. mutans*^WT^, the lack of *S. gordonii* AI-2 signalling results in decreases in the dual-species biofilm mass [65]. In ΔluxS *S. mutans* strains, ABC transporters are downregulated [66]. QQ inhibition of *S. mutans* biofilms has been achieved using substances derived from sources as diverse as natural products, bacterially produced antimicrobials, synthetic materials, and human saliva (Figure 3).

#### 4.1.1. CSP/ComABCDE QS Pathway in *S. mutans*

Over 20 years ago, a competence-stimulating peptide export system based on an ATP binding cassette (ABC) transporter, ComAB, was described for *S. mutans* [72]. Soon after, a QS feedback loop, ComCDE was reported [73]. The system follows the Gram-positive model outlined in Section 2.2. The comC, comD and comE genes express, respectively, ComC, the precursor of the competence stimulating peptide (CSP), ComD, a transmembrane histidine kinase signal transducer and ComE, a response regulator protein [73]. ComC is first truncated by the ABC transporter into a 21 amino acid protein, then further processed by an extracellular membrane-bound protease, SepM, into CSP, an 18 amino acid AI [74]. CSP binding of ComD results in ComE phosphorylation which causes the transcription of an alternate sigma factor, called ComX, from the comX gene to induce an *S. mutans* phenotype that is genetically competent [73]. The ComABCDE system is also important for the initiation of biofilm formation [75], and the expression of bacteriocins from, for example, the bacteriocin expressing genes nlmA and nlmC [76].

Additionally, it is known that the ComABCDE system can be bypassed because both competence and bacteriocin production can be activated independently through a response regulator called HdrR [60]. HdrR is expressed from the hdrRM operon which also expresses HdrM, an inhibitor protein that blocks the action of HdrR, thus providing alternative regulation of the conversion to genetically competent and bacteriocin-producing phenotypic forms [60].

#### 4.1.2. XIP-ComRS QS Pathway in *S. mutans*

The ComABCDE system is networked with the other main feedback loop for intercellular communication between cells in *S. mutans* biofilms which involves the comX inducing peptide (XIP), expressed as the percussor ComS [77]. A significant difference between the two pathways, is that in addition to transmembrane transporters, cell lysis appears to be a major mechanism for discharging the AI extracellularly [77]. XIP is then imported back into the cell by a membrane oligopeptide permease, OppD [78], where, in many streptococcal species including *S. mutans*, it binds the response regulator ComR [79]. The ComR-XIP complex acts as a transcriptional activator of the genes comS, comR and comX in a feedback loop, with transcription of comX resulting in genetic competence [61]. Current work in other streptococci has identified key amino acids in the interaction between ComR and XIP which results in the formation of an active complex [80].

#### 4.1.3. PdrA-WGK QS Pathway in *S. mutans*

In 2021, the *S. mutans* wgk operon was shown to be regulated by a short hydrophobic AIP (SHP) and a response regulator called PdrA. SHP is exported out of the cell by the transmembrane exporter PptAB and reimported by an Opp transporter. Activation of the operon results in the production of a peptide called tryglysin B, a 7-amino acid peptide containing the sequence Try-Gly-Lys. Tryglysin B inhibits several oral streptococci including *S. mitis*, *S. oralis*, *S. sanguinis*, and to a lesser extent *S. gordonii*. Tryglsin B is also autoinhibitory; however, *S. mutans* recovers from this inhibition by 24 h [64]. Detailing the PdrA-WGK pathway has further helped understand virulence in *S. mutans* by showing how it can inhibit competing oral streptococci.

#### 4.1.4. QQ in *S. mutans* by Plant Products

Inhibition of *S. mutans* biofilm formation, in association with downregulation of comDE, has been associated with the treatment of biofilms with several plant products. These include baicalein (5,6,7-trihydroxyflavone), obtained from *Scutellaria baicalensis* and *Scutellaria lateriflora* [26], extracts from *Rhodiola rosea* [81], and *Emblica officinalis*, the Indian gooseberry [24]. In the case of baicalein and *E. officinalis*, biofilm hydrophobicity, important to bacteria-carbohydrate interactions, was shown to decrease. Baicalein treatment also resulted in the downregulation of comX and luxS, illustrating the potential to disrupt both intraspecies and interspecies cellular communication [26]. Combining plant products with common antimicrobials and fluoride has been tested. When chlorhexidine and fluoride were coupled with trans-cinnamaldehyde obtained from cinnamon, downregulation of comADE, comX and luxS was observed, associated with biofilm inhibition which complemented the action of chlorhexidine [82].

#### 4.1.5. QQ in *S. mutans* by Microbial Products

*S. mutans* competitively coexists in oral biofilms with several other species, the products of which can disrupt QS in *S. mutans*. *S. gordonii* and *S. sanguinis* can not only be inhibited by *S. mutans* produced tryglysin B, but conversely, can disrupt ComRS and ComE signalling in *S. mutans*, causing the degradation of *S. mutans* biofilms [67]. Walkmycin C, an antibiotic produced by *Streptomyces* strain MK632-100F11 [83], targets *S. mutans* comDE [84]. Fructanase, an enzyme produced by *Streptococcus salivarius*, [85] adversely affects *S. mutans* competence and bacteriocin production [59]. The products of probiotic *Lactobacillus* species can downregulate *S. mutans* comD [86], while antimicrobials produced by *Lactiplantibacillus* species target *S. mutans*, comA and comX [57]. Thus, there appear to be many opportunities for bacterial products to be trialled as anticaries agents. However, it must also be recognized that a complex competitive bacterial network is operational in the oral cavity, the implications of which are far from completely understood.

#### 4.1.6. QQ in *S. mutans* by Synthetic Molecules

Synthetic QQ molecules are a further avenue of research in caries prevention. A bicyclic amine of the quinuclidine class was found to downregulate the bacteriocin-producing genes nlmA and nlmC and inhibit genetic competence in *S. mutans*. Inhibition occurred via non-competitive binding of the peptidase domain of ComA, causing allosteric changes at the enzyme active site [76]. Other synthetic compounds include small peptide analogues of XIP, capable of disrupting XIP binding to ComR [56], and nano-quercetin particles used in conjunction with photodynamic therapy, causing downregulation of comBCDE [87]. Finally, human saliva contains a naturally occurring mucin, MUC5B, able to inhibit *S. mutans* biofilm formation. The mechanism operates through the linkages and structures of the O-linked glycans forming the mucin. It affects both the CSP and XIP pathways [34].

### 4.2. QS and QQ in Candida albicans and Dual Species S. mutans-C. albicans Biofilms

Farnesol is a lipophilic, QS molecule that is secreted into the extracellular environment by *C. albicans* [71]. At high cell density, farnesol suppresses an enzymatic pathway, the Ras1-Cdc35-PKA-Efg1 pathway, that permits hyphal transformation important to the virulence of this species [88]. Farnesol has a sesquiterpene structure similar to that of dodecanal which also is able to repress the same pathway and inhibit the transformation to the hyphal form [88]. Another compound, the tryrosine derivative tyrosol, like farnesol, is a QS molecule in *C. albicans*, and also like farnesol, it can inhibit *C. albicans* biofilm formation [89].

However, at low concentrations, farnesol can promote rather than inhibit biofilm formation important in cariology by stimulating *S. mutans* growth [90]. In this context, farnesol secreted by *C. albicans* can augment biofilm growth in dual-species biofilms of *C. albicans* and *S. mutans* by increasing the expression of *S. mutans* glucosyl transferases [90]. Glucosyl transferases increase sugar metabolism in *S. mutans*, resulting in the secretion of a glucan-rich adherent plaque [91], favourable to *C. albicans* [90]. This interkingdom relationship between *S. mutans* and *C. albicans* appears to be finely balanced, but symbiotic. It permits *C. albicans* to grow well, when unaccompanied it would not, and at the same time, it increases the virulence of *S. mutans* [90]. It has been shown that this relationship induces the transcription of *S. mutans* comC and comX, implicating the AIs CSP and XIP, genetic competence and bacteriocins in the mechanism [92]. The involvement of *S. mutans* QS networks in these dual-species biofilms was confirmed by a later study which also reported changes to comC expression [93].

It is noteworthy, however, that at high farnesol concentrations in S. *mutans-C. albicans.* biofilms, growth is inhibited [90], providing opportunities to employ QQ against cariogenic biofilms. Indeed, farnesol has been used in combination with fluoride and plant products (a flavonoid called myricetin and flavonoid metabolites) to reduce *S. mutans-C. albicans* cell numbers [94]. Other plant products have been used to inhibit dual species *S. mutans-C. albicans* biofilms. Both curcumin [25] and the plant derivative thymol [95] interfere with *S. mutans* CSP QS via inhibition of comCD expression. This impairs biofilm development, an effect which, in the case of curcumin, is stronger for *S. mutans* than for *C. albicans*. Certain drugs have also been used as QS inhibitors in this situation. Thiazolidinediones are used in the treatment of diabetes [96]. A related compound, thiazolidinedione-8 has been shown to be effective against QS in *S. mutans-C. albicans* biofilms, with the effect being strongest against the *C. albicans* component [97].

## 5. QS and QQ in Periodontitis

The oral cavity is known to harbour more than 700 to 800 diverse bacterial species [57]. A complex interplay exists among this large group of bacteria during health and disease. The microbial interaction is not merely limited to an interaction between healthy and pathogenic bacteria, but also involves a positive feedback loop between the bacteria and the host immune-inflammatory response [20]. Thus, during eubiosis and dysbiosis, the interaction is multi-dimensional [20]. Some of the bacterial by-products generated by one group of bacteria may be sensed as growth promoters by other groups of bacteria, while, for another group of bacteria, the same by-products may be inhibitory. Several periodontal pathogens produce AI-2 molecules which may be involved in cell–cell signalling within these biofilms [20]. AI-2 signalling influences the response to host-generated stress, environmental stress, the acquisition of iron and hemin and the development of periodontal biofilms [20]. Sites with progressive periodontitis can demonstrate increased gene expression related to periodontal pathogenesis. This particularly applies to the oxidative stress response, ferrous iron transport, amino-acid transport, and the synthesis of lipopolysaccharides [98]. Non-progressive sites may exhibit stable gene expression over the follow-up [98]. 

Several periodontal bacteria including *Porphyromonas gingivalis*, *Treponema denticola*, *Tannerella forsythia*, *Prevotella intermedia* and *Fusobacterium nucleatum* have been studied for their QS properties and may serve as potential targets for QQ [99]. Because biofilm formation and various virulence properties of periodontal biofilms are controlled by QS, several possibilities exist to interfere with QS signalling systems, and numerous novel attempts at QQ have been made.

The majority of the research investigating QS among bacteria associated with periodontitis is focused on *P. gingivalis*, a putative bacterium which is considered a “keystone pathogen” in periodontitis [100]. Its presence in sufficient quantities is known to convert the entire microbiota into dysbiosis [20,101]. The red-complex species, *P. gingivalis*, *T. denticola* and *T. forsythia*, which are frequently isolated from sites with active periodontitis [102], form an important group of pathogens evaluated for their QS behaviour. *F. nucleatum*, often considered as the bridge organism responsible for the coaggregation between early commensal bacteria and late pathogenic bacteria [103], has also been studied for QS properties, and subsequently as a target for QQ in the treatment of periodontitis. While much of the QS and QQ research in periodontitis is in *in vitro* models, a limited number of *in vivo* models exist, especially in murine species.

### 5.1. QS Interactions among Periodontal Pathogens

Quorum sensing also influences the complex intraspecies and interspecies interactions among various pathogens. In 2001, strains of *P. intermedia*, *F. nucleatum*, and *P. gingivalis* were reported as having AI-2-like activities [99]. The authors also reported the inability of these pathogens to produce homologues of AHL [99]. However, recent *in vitro* evidence suggests that periodontal pathogens produce short-chain AHL molecules [104]. In subgingival biofilms, there are reports that the AI-2 of *F. nucleatum* promoted biofilm formation and helped in inter-species coaggregation between *F. nucleatum* and each of the red complex species [105]. Similarly, *F. nucleatum* AI-2 resulted in increased biofilm formation of *S. gordonii* and *S. oralis* [106], and enhanced the attachment of *F. nucleatum* to these streptococci [105]. The strongest interaction existed between *F. nucleatum* and *S. mitis* and the addition of other bacteria may interfere with this co-adherence [107]. *Aggregatibacter actinomycetemcomitans* serotypes b and f were shown to aggregate with *F. nucleatum*, mediated by QS through an O-polysaccharide [108].

*Eikenella corrodens* can express a gene that is essential for the production of AI-2 molecules and the presence of AI-2 molecules can influence the formation of *E. corrodens* biofilms [109]. The efficiency of *E. corrodens* biofilm formation in the presence of AI-2 molecules was 1.3 times greater than in its absence [109]. In dual-species biofilms grown on artificial saliva, *Aggregatibacter actinomycetemcomitans* activated the QS regulon of *S. mutans* [110]. Biofilm formation and the virulence of *Aggregatibacter actinomycetemcomitans* are controlled by the release of AI-2. However, gene expression following the release of AI-2 molecules is controlled by the release of the QseBC two-component system as evidenced in an *in vivo* mouse model [111].

Several *in vitro* and *in vivo* models have been explored to understand the network of QS in periodontitis. However, due to the complex interplay of various bacteria involved in periodontitis, more research work is essential to decipher the role of QS in the pathogenesis of periodontitis.

### 5.2. QQ Compounds in Periodontitis

#### 5.2.1. Furanone Compounds and Combinations with D-Ribose

Furanones, including 2(5H)-furanone, have been explored as potential QQ agents against periodontal pathogens [112]. This is because various furanones are similar to N-AHL in terms of their chemical structure. Additionally, they inhibit biofilm formation without the development of AMR [112].

Brominated furanones show promise as QQ compounds. Monocyclic brominated furanones produced by the red algae *Delisea pulchra*, have antibiofilm properties associated with the modulation of QS genes [113]. Synthetic bicyclic brominated furanones have applications against *F. nucleatum*, *P. gingivalis*, and *T. forsythia* biofilms, as evidenced by reductions in biofilm biomass and thickness, [114]. The further potential of the use of brominated furanones against periodontal pathogens is shown by their ability to reduce *P. gingivalis* biofilm biomass, causing the scattering of *P. gingivalis* cells [115]. The authors reported that brominated furanones showed concentration-dependent effects and had potential as an antimicrobial treatment for periodontitis [115].

Brominated furanones have also been combined with D-ribose to evaluate their anti-AI-2 effect on coaggregates of *P. gingivalis* and *F. nucleatum* in an *in vivo* murine model [44]. A reduction in the periodontal breakdown, as evidenced by reduced alveolar bone loss and decreased bacterial infection of the periodontal tissues was observed [44]. Similarly, the combination of a brominated furanone [116] and D-ribose in a murine model showed reduced bone loss and decreased levels of *P. gingivalis* [117]. Furthermore, (5Z)-4-bromo-5-(bromomethylene)-2(5H)-furanone and D-ribose, can inhibit AI-2 induced *F. nucleatum* coaggregation, with individually, all three red complex pathogens [105]. These same QS inhibitors were also shown to interfere with early oral biofilm colonization by streptococci, again via the inhibition of *F. nucleatum* AI-2 [106].

#### 5.2.2. D-Galactose

D-galactose also has QQ properties. The results of crystal violet and confocal microscopy assays showed that D-galactose inhibited the activity of AI-2. This resulted in decreased formation of *F. nucleatum*, *P. gingivalis*, and *T. forsythia* biofilms [118]. D-galactose also inhibited the coaggregation of *A. actinomycetemcomitans* serotypes b and f with *F. nucleatum* [108].

#### 5.2.3. Natural Products

Several plant-derived products and phenols, such as coumarin and green tea extracts, have been explored for their QS inhibition properties. Coumarin, inhibited biofilm formation at various stages and downregulated the expression of *P. gingivalis* biofilms and luxS genes. Extracts from green tea and its main component epigallocatechin-3-gallate (EGCG) inhibited the growth and adherence of *P. gingivalis* biofilms and also downregulated various genes responsible for the virulence of *P. gingivalis* [119]. While green tea extract and ECCG were able to inhibit AI-2 in a *Vibrio harveyi* model, their role in QQ in *P. gingivalis* biofilms is yet to be verified [119].

#### 5.2.4. Synthetic Analogues of AHL

Several synthetic analogues of AHLs have been shown to inhibit *P. gingivalis* biofilm formation with three of them able to significantly decrease the number of biofilm cells [120]. A less well-formed biofilm structure, as demonstrated by confocal microscopy, was observed. Modifications to AHL were made by the introduction of varying numbers of either alcohol or amine groups into the acyl groups, which also contained altered numbers of carbon atoms.

## 6. Coating Surfaces of Dental Implants with QS Inhibitors

**Figure 4:**
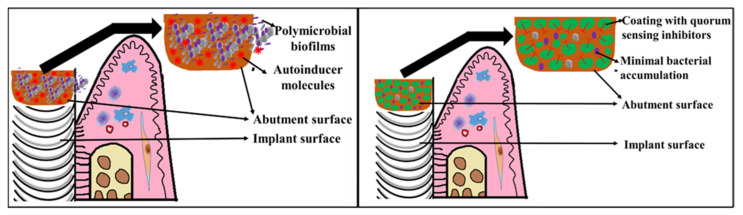
Implant abutment surfaces are common areas where bacteria accumulate, resulting in the induction of quorum sensing through the release of autoinducer molecules. Robust virulent biofilms are formed which may cause peri-implantitis. Implant abutment surfaces can be coated with QS inhibitors resulting in quorum quenching. This inhibits biofilm formation.

Every year, many medical devices including dental implants, prostheses, bone plates, bone grafts and membranes are placed within the mouth [121,122,123]. The abutment surfaces of dental implants typically get covered by biofilms, leading to medical-device-associated infections such as peri-implantitis [124,125]. Thus, device removal or additional surgeries are required [126]. Recently, alternative approaches including the use of lasers and photobiomodulation have reported significant adjunctive clinical benefits compared to conventional scaling and root debridement in the treatment of peri-implantitis [127]. QQ may provide a solution to this problem. Coating the dental implant abutment surfaces with QS inhibitors may result in reduced biofilm formation (Figure 4).

Microparticles coated with QS inhibitors (calcium-binding polymer-coated poly(lactide-co-glycolide) have been placed on hydroxyapatite surfaces to prevent *S. mutans* biofilm formation [128]. Because most dental hard tissues are made up of hydroxyapatite, these inhibitors may have the potential for research in other dental disciplines.

Pre-treatment of titanium discs with D-arabinose for three minutes has been shown to inhibit the formation of both monospecies biofilms of *S. oralis*, *F. nucleatum* and *P. gingivalis* and a biofilm consortium of the same three bacterial species [129]. The authors also suggested that D-arabinose had initial anti-adhesive properties against biofilm-forming bacteria and sustained inhibitory activity against AI-2 [129]. The research concluded that D-arabinose may be used to prevent peri-implant mucositis and peri-implantitis [129].

QS inhibition has been demonstrated in other fields. Agents involved in urinary tract infections, *Pseudomonas aeruginosa* and *Serratia marcescens*, can be inhibited by coating thiazolinyl-picolinamide-based palladium (II) complexes on urinary catheters [130]. Similarly, heteroleptic pincer palladium (II) complex coated orthopaedic implants resulted in QQ and inhibited the development of *Acinetobacter baumannii* biofilms [131]. However, the possible application of this approach to peri-implantitis may pose problems. An important differentiating factor in urinary tract and orthopaedic infections versus peri-implantitis is that the former are mostly due to single species or dual-bacterial species. Conversely, peri-implantitis is associated with a high bacterial diversity [132,133].

## 7. Conclusions, Limitations, and Future Directions

Improving the knowledge base of QS and QQ mediators and mechanisms has the potential for the development of antibiofilm treatments. Some areas of dentistry are underrepresented in the QS/QQ literature, and the focus of this review aligns with the main areas of research. To date, in cariology and periodontology, interfering with QS signals has been effective predominantly in *in vitro* research to decrease biofilm formation and virulence. The switch to *in vivo* focused research would be the next step in realizing some of the clinical potential for QS inhibitors from diverse natural and synthetic substances, including carbohydrates, and autoinducer analogues. Such research would reveal the effects of QQ substances on naturally occurring polymicrobial biofilms. Many of the *in vitro* studies presented in this review have been performed on monospecies biofilms, particularly with respect to dental caries, where *S. mutans* biofilms predominate. Periodontal QQ research is based on *P. gingivalis* or a consortium of bacteria including, amongst others, *P. gingivalis*, *T. forsythia* and *F. nucleatum*. However, current evidence indicates that polymicrobial synergy and dysbiosis of the entire microbial community are involved in dental diseases. Additionally, naturally occurring biofilm infections are a result of complex interactions between multiple pathogenic bacteria, host-compatible bacteria, and the host’s immuno-inflammatory cascade. Thus, whether the positive QQ results outlined in this review can be replicated in naturally occurring microcosms remains to be ascertained.
